# Analysis of the Fracture Resistance of Buildings on Composite Foundations with Horizontal Reinforcement Crossing Normal Faults

**DOI:** 10.3390/s26010090

**Published:** 2025-12-23

**Authors:** Jiankang Tian, Jianyi Zhang, Haonan Zhang, Yonghua Zhang, Hongjuan Chen, Shuai Wang, Yunfan Zhou, Ziyi Feng

**Affiliations:** 1 College of Geological Engineering, Institute of Disaster Prevention, Sanhe 065201, China; tjk0624@163.com (J.T.); wangshuai51881016@163.com (S.W.); zyf3703@163.com (Y.Z.); fziyii@163.com (Z.F.); 2 Key Laboratory of Earthquake Engineering and Engineering Vibration, Institute of Engineering Mechanics, China Earthquake Administration, Harbin 150080, China; zhn1027iem@163.com; 3 North China Geological Exploration Bureau Comprehensive Survey Team, Sanhe 065201, China; 15512637306@163.com; 4 Institute of Geophysics, China Earthquake Administration, Beijing 100081, China; chenyu94@163.com

**Keywords:** earthquake surface rupture, geogrid, physical model test, precursor indicator, differential deformation

## Abstract

To investigate the performance of horizontally reinforced composite foundations in resisting surface rupture of normal faults, this study designed and conducted a series of physical model tests. A systematic comparative analysis was performed on the fracture resistance of sites with three-layer sand, five-layer sand, and three-layer clay geogrid horizontally reinforced composite foundations under 70° normal fault dislocation. The results indicate that significant changes in earth pressure serve as a precursor indicator of fault rupture, and their evolution process reveals the internal energy accumulation and release mechanism. Increasing the number of geogrid layers significantly enhances the lateral confinement of the foundation, resulting in a narrower macro-rupture zone located farther from the structure in sand sites, and promotes the formation of a step-fault scarp deformation mode at the surface, which is more conducive to structural safety. Under identical reinforcement conditions, the clay site exhibited comprehensively superior fracture resistance compared to the sand site due to the soil cohesion and stronger interfacial interaction with the geogrids, manifested as more significant deviation of the rupture path, and lower microseismic accelerations and structural strains transmitted to the building. Comprehensive analysis confirms that employing geogrid-reinforced composite foundations can effectively guide the surface rupture path and improve the deformation pattern, representing an effective engineering measure for mitigating disaster risk for buildings spanning active faults.

## 1. Introduction

**Figure 1:**
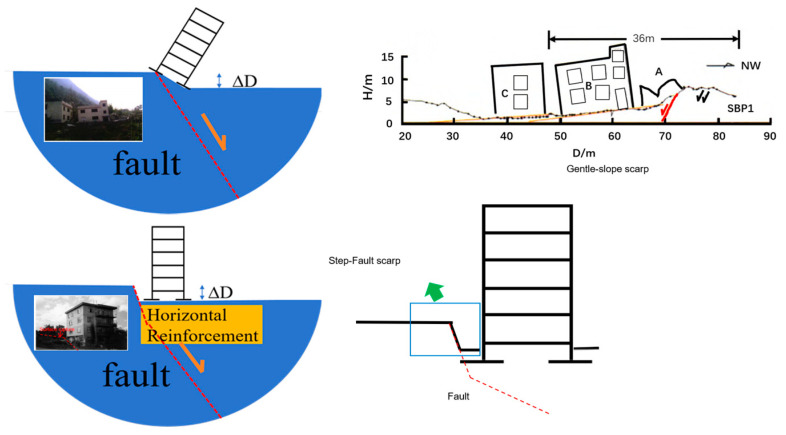
Schematic diagram of resisting significant differential surface deformation resulting in a Step-Fault scarp (adapted from Anastasopoulos, I [10] and Guo [11]).

Recent strong seismic events have demonstrated that surface rupture and the resulting significant differential deformation caused by active fault displacement pose a serious threat to buildings and structures adjacent to the rupture zone [1,2,3,4,5]. For instance, during the 2008 Wenchuan earthquake, the building cluster in Xiaoyudong Town, which shared identical structural design and construction quality, was subjected to fault displacement. Structures within the zone of differential deformation almost completely collapsed, while adjacent buildings sustained relatively less damage. This indicates that fault displacement and the induced differential deformation were the primary causes of structural destruction [6]. In the 2023 Turkey M7.8 earthquake, two reinforced concrete buildings located within the large deformation zone where the fault reached the surface were completely destroyed. Warehouses and farmhouses along the rupture zone also experienced widespread collapse due to differential displacement, further revealing the extremely high disaster risk associated with significant differential deformation triggered by surface rupture [7]. In the 2025 Myanmar M7.9 earthquake, an office building was severely damaged due to the deformation scarp from surface rupture, whereas nearby buildings unaffected by the large deformation sustained lighter damage [8]. Collectively, these cases indicate that for structures located within the surface rupture zone, in addition to the direct damage caused by fault displacement, the significant differential surface deformation induced by fault movement is another key factor contributing to their damage (Figure 1). It is noteworthy that some cases show that an appropriate structure-foundation system can effectively mitigate the impact of fault displacement.

For example, during the 1999 İzmit earthquake in Turkey, a four-story reinforced concrete building with a basement survived a vertical fault displacement of 2.3 m. The relatively high stiffness of the basement altered the surface deformation pattern from a gentle-slope scarp to a step-fault scarp, effectively protecting the superstructure. Similarly, in the same earthquake, a building employing a box foundation exhibited only fine cracks under 2.1 m of normal fault displacement. The reason was that the building owner had considered foundation reinforcement during the design phase, using the box foundation in a manner analogous to horizontal reinforcement, demonstrating the potential of horizontal reinforcement in regulating deformation and protecting the superstructure [9,10].

With more in-depth statistical analysis of fault-induced seismic damage by researchers [12,13,14,15,16], various geotechnical mitigation strategies for seismic surface fault rupture have consequently been proposed [17]. Further research has confirmed that targeted ground improvement can divert the fault rupture path away from structures and improve the pattern of large surface deformation, thereby effectively reducing damage to the superstructure [18]. To investigate which types of foundation-soil systems can effectively mitigate damage to superstructures caused by significant differential surface deformation. Scholars have conducted explorations from two aspects: To systematically evaluate the effectiveness of different mitigation strategies and reveal their underlying mechanisms, numerous scholars have employed numerical simulation methods for investigation [19,20,21,22,23,24]. For instance, Yao [25] based on numerical simulations, analyzed the improvement of fault rupture path by ground improvement methods such as rigid foundations. Habib Rasouli [26] established an anti-fracture model for a composite foundation with geosynthetics, which can significantly reduce foundation strain response, indicating the potential of such composite foundations in protecting the superstructure. Bray [27] proposed using reinforced fill to extend the differential deformation zone, thereby reducing tensile strain at shallow foundations, offering a new perspective for engineering construction. Numerical simulation methods provide an effective analytical tool for geotechnical mitigation strategies against seismic surface fault rupture, but they still have limitations in simulating overburden-foundation-structure interaction, large soil deformation, and complex failure processes. Therefore, it is necessary to establish physical model tests to investigate the mitigation mechanisms of composite foundation treatments for structures spanning faults and to analyze the overall failure process where overlying soil rupture and large surface deformation affect the superstructure.

Researchers have proceeded to establish relevant physical model tests. Recent studies by Rasouli and Fatahi et al. [28,29,30,31,32,33,34,35] and Yang et al. [36,37,38] have fully demonstrated the significant potential of geosynthetics in resisting surface rupture and regulating deformation patterns, providing the core theoretical foundation for this study. However, current research still has limitations in terms of scale, complexity, and observation methods. Compared to the small-scale models with single homogeneous soil commonly used by researchers, there is an urgent need to establish large-scale physical models employing various soil types to simulate more realistic geological conditions. Furthermore, existing physical models lack monitoring of the deep overburden response during fault development, making it difficult to obtain precursors of deep fault activity and to establish a reasonable relationship between deep-seated information and surface-monitored disasters. Furthermore, existing physical models lack monitoring of the deep overburden response during fault development, making it difficult to obtain precursors of deep-seated fault activity and establish a rational correlation between deep-seated information and surface-monitored disaster phenomena.

To address this, this study established a large-scale, complex geomechanical model for both sand and clay sites, overcoming the limitation of relying primarily on macro-deformation analysis. By integrating displacement meters, earth pressure cells, and accelerometers, it verified the time-lag phenomenon between deep stress changes and surface rupture. It captured the dynamic response at the instant of rupture, providing crucial “precursor” information for real-time fault monitoring systems. It established a comprehensive analytical framework linking “fault rupture—foundation response—structural performance,” thereby deepening the understanding of soil-structure interaction mechanisms. The study revealed the evolution patterns of internal dynamic responses within the foundation during fault displacement, offering novel data for mechanistic understanding.

## 2. Design of the Model Test Scheme for Horizontally Reinforced Composite Foundations Across Normal Faults

**Figure 2:**
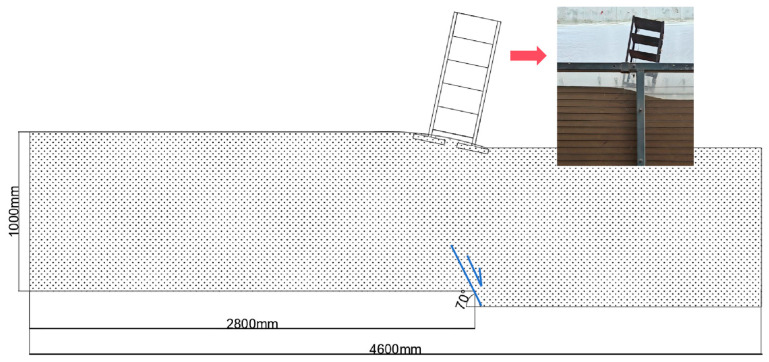
Schematic diagram of the model container.

This test positioned the building at the most unfavorable location within the strong surface deformation zone. By incorporating an anti-fracture design with geogrids placed in key soil layers, it investigated the fracture resistance performance of reinforced soil foundations for buildings spanning active faults. A geometric similarity ratio of *N* = 20 was adopted to simulate the dislocation of a 70° normal fault within the model container, with displacement controlled in stages using a hydraulic jacking system (Figure 2). The tests compared the anti-fracture effectiveness of the geogrids under varying numbers of reinforcement layers, different layer spacings, and in different soil types (sand/clay). Dynamic and deformation responses were monitored via sensors installed within the soil mass, on the geogrids, and on the structure. The aim was to elucidate the corresponded working mechanism and the resulting mitigation effectiveness of the reinforced soil foundation-structure system under fault movement.

To ensure the scientific validity and repeatability of the physical model tests, the entire experimental process followed four main stages: model preparation, model construction, loading, and data acquisition. First, the geometric similarity constants were determined, and the composite foundation scheme was designed based on the prototype structure and site conditions. Subsequently, the mechanical properties of the test materials were analyzed, and all sensors were calibrated. Following the composite foundation scheme, the test overburden was constructed. During the layered filling process, horizontal geogrids were installed, and sensors were embedded at predetermined depths. After setup, loading was performed by precisely controlling the fault dislocation angle and rate, while simultaneously observing phenomena and collecting data.

### 2.1. Model Test Apparatus and Working Principle

The model test apparatus, as shown in the figure, consists of four main components: the model container, the power system, the monitoring sensors, and the data acquisition system. This test simulated a normal fault with a dip angle of 70°. The front and rear sides of the model container were constructed from high-strength transparent acrylic panels to facilitate observation of the test phenomena, while the left and right sides were made of high-strength steel plates. The base of the model container was divided into a stationary end (passive plate) and a movable end (active plate); fault movement was simulated by jacking the movable plate. Four hydraulic cylinders provided the power required for fault dislocation. They were controlled by a console to apply displacement at a rate of 2 mm/s, with a total displacement of 100 mm.

### 2.2. Model Preparation for the Tests

#### 2.2.1. Similitude Constant

Based on the failure characteristics of buildings crossing faults during strong earthquakes such as the Chi-Chi and Wenchuan events [1,6], and drawing on previous physical modeling experience of dip-slip faults [28,36], this test design aims to replicate the seismic damage mechanisms under typical engineering conditions. In accordance with the definition of overburden thickness in building seismic design codes, and after comprehensively considering the size effect of the model container, boundary conditions, and material similitude limitations, a geometric similarity ratio of 1:20 was ultimately determined (As shown in Table 1 and Table 2). Accordingly, a model soil layer with a depth of 1000 mm was constructed for the tests, precisely simulating a 20 m thick prototype overburden environment to meet the research needs for studying the response to 70° dip-angle fault displacement in both sand and clay sites.

#### 2.2.2. Overburden Soil Layer

Based on the physical property indices of the in situ soil, such as density and water content, suitable materials were selected and processed to ensure consistency in the mechanical properties of the soil samples. Prior to testing, clay samples were first collected from the natural environment and carefully sieved to remove impurities, thereby obtaining purified clay samples. For the sand samples, standard testing sand that met specifications was used directly. To ensure that the soil layers in the test maintained the same relative density, a quantitative compaction method was employed, whereby a 150 mm layer of uncompacted soil was repeatedly compacted and leveled to achieve a 100 mm thick compacted layer. This process was repeated until the total compacted height of the soil layer reached 1000 mm.

#### 2.2.3. Building Model

**Figure 3:**
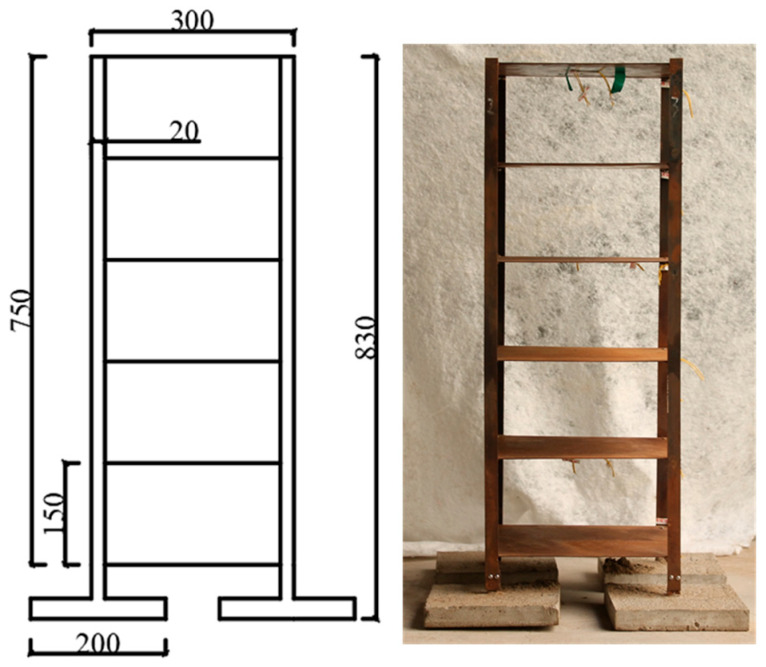
Building model diagram.

**Figure 4:**
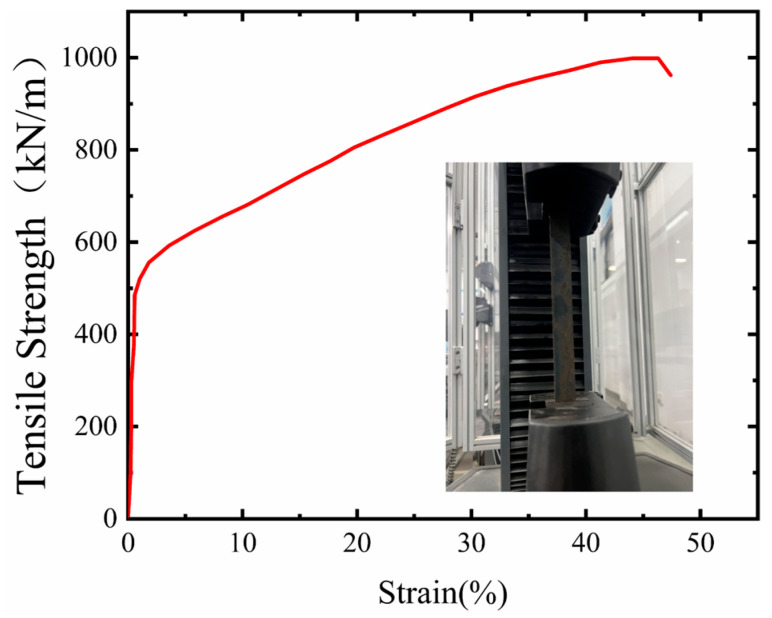
Steel tensile test diagram.

The dimensions, materials, and structural design of the building model underwent rigorous similarity analysis to ensure its behavior in the model tests accurately reflects the physical and mechanical properties of a real building. The configuration of the building model is illustrated in the accompanying figure. The structure was a 6-story steel frame model with a story height of 150 mm. The floor slabs were square steel plates measuring 300 mm × 300 mm, and the isolated column foundations were square concrete model blocks of 200 mm × 200 mm × 30 mm, with a compressive strength of 3.75 MPa (As shown in Figure 3 and Figure 4).

#### 2.2.4. Horizontal Reinforcement

**Figure 5:**
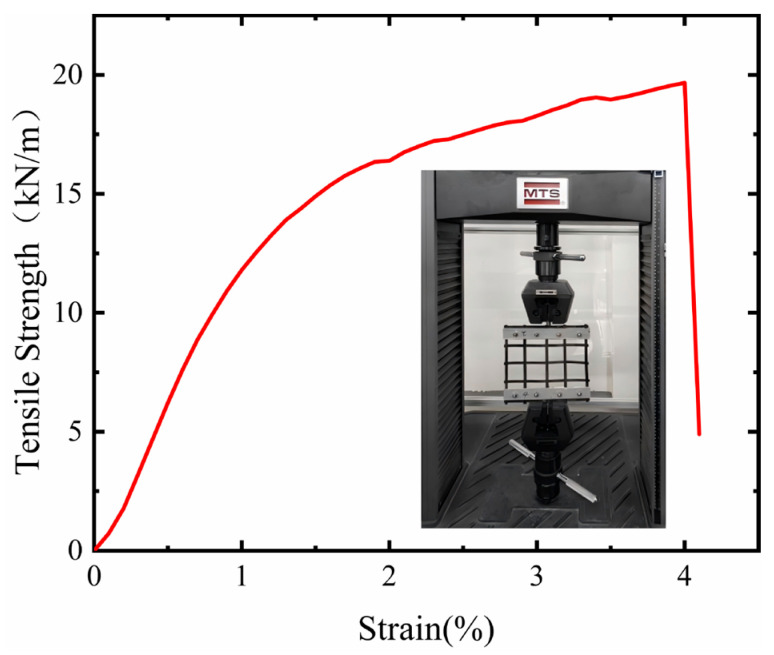
Geogrid tensile test diagram.

Based on performance tests of geogrids by relevant researchers [39,40,41,42,43], this test employed a biaxial steel-plastic geogrid, with both the longitudinal and transverse ribs of a single grid measuring 60 mm in length. The structure of the geogrid is illustrated in the accompanying figure. For the tensile test of the geogrid, this study utilized a microcomputer-controlled electronic universal testing machine and conducted a uniaxial tensile test using the multi-rib method. The test results are shown in Figure 5.

The geogrid selected for the tests exhibited three distinct stages during the tensile process. The initial stage was characterized by low strength and small strain, where both the tensile strength and geogrid strain were minimal. This was followed by a high-strength, large-strain stage, where increasing strength led to a gradual rise in geogrid strain. The final stage was the fracture stage, where exceeding a certain tensile strength limit initiated the failure of the geogrid’s internal structure, causing a rapid decrease in tensile strength. Specifically, the steel-plastic biaxial geogrid achieved a tensile strength of 19 kN/m at a strain rate of 4%. These results fully demonstrate that this geogrid possesses high tensile strength and favorable deformation characteristics, meeting the mechanical performance requirements for the test materials (as shown in Figure 5).

### 2.3. Layout of Test Instruments

**Figure 6:**
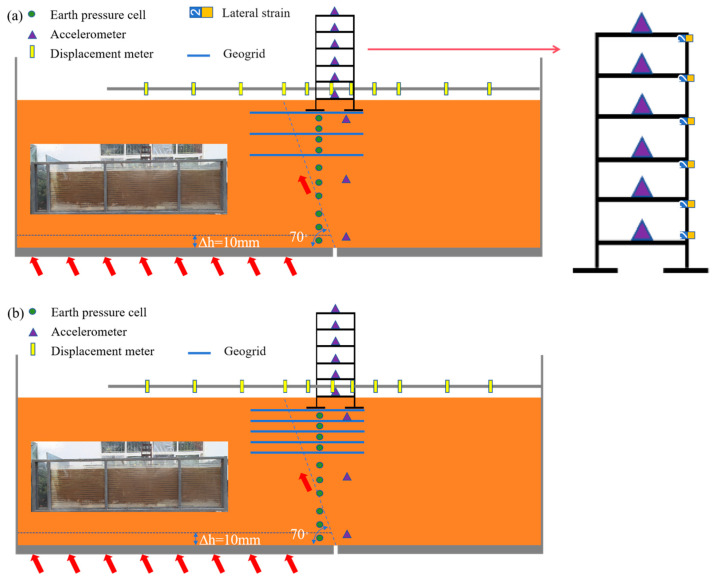
Layout diagram of test instruments: (**a**) three-layer geogrid (**b**) five-layer geogrid.

**Figure 7:**
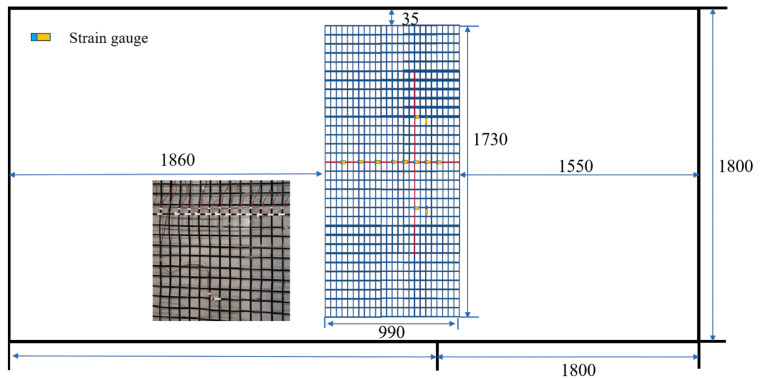
Top view of geogrid arrangement and strain gauge layout on the geogrid. (Unit: mm).

The detailed test scheme is shown in Table 3. Prior to installation, all sensors, including earth pressure cells, accelerometers, and displacement meters, underwent rigorous static and dynamic calibration to ensure measurement accuracy and data reliability throughout the test duration. As shown in Figure 6 and Figure 7, for real-time monitoring of the dynamic response and earth pressure changes within the overburden soil, accelerometers and earth pressure cells were installed at key locations where the fault traverses the soil mass, to capture in real time the propagation patterns of dynamic response and the pressure evolution process at different depths [44]. A set of strain gauges were arranged along the length of the geogrid to accurately analyze the strain distribution patterns and stress transfer mechanism of the geogrid structure under load. Additionally, accelerometers and strain gauges were installed on each story of the building to detect its dynamic and stress responses during the test. Finally, by utilizing the multi-source data collected by the acquisition system, a comprehensive dynamic response analysis of the interaction among the overburden, reinforced foundation, and building was conducted [45,46,47,48]. Relevant sensor parameters are presented in Table 4.

## 3. Comparative Experimental Analysis of Sites and Building Seismic Damage Under Normal Fault Bedrock Dislocation

The experimental data for the test on the horizontally reinforced composite foundation across a high-angle normal fault primarily encompassed earth pressure, acceleration of the overburden and the structure, structural strain, and geogrid strain. During each test, data were collected at every 10 mm of dislocation, with each interval recorded as a working condition, resulting in a total of 10 working conditions. The test process could be divided into three stages based on fault propagation (initial stage, minor basal dislocation stage, and final rupture stage), One representative working condition from each stage was selected for comprehensive comparative analysis.

### 3.1. Comparative Analysis of Sand Site with Three-Layer Horizontal Reinforcement Versus Sand Site with Five-Layer Horizontal Reinforcement

#### 3.1.1. Comparative Analysis of Fault Rupture

**Figure 8:**
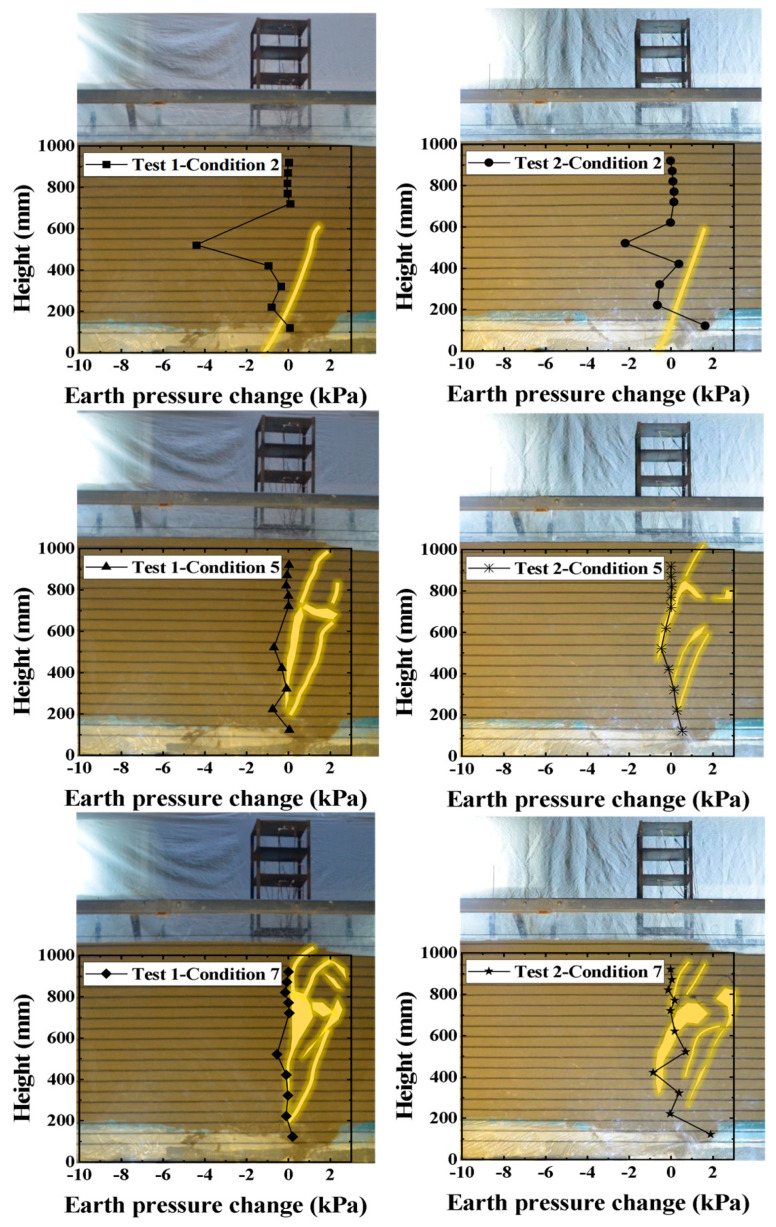
Diagram of earth pressure variation within the overburden and the development pattern of the rupture trace (Comparison of Test 1 and Test 2).

As shown in Figure 8, which presents a series of front views and the corresponding internal earth pressure changes for Test 1 and Test 2 at bedrock dislocation amounts of 2 cm, 5 cm, and 7 cm, the red dashed line depicts the fault rupture path, while the black solid line represents the variation in earth pressure within the soil mass. The figure reveals a close relationship between earth pressure and the soil failure zone, where changes in earth pressure precede and predict the development of visible rupture, unveiling the mechanism of energy accumulation and release during fault displacement. Significant earth pressure changes occur even during minor soil cracking, whereas when a larger failure zone develops, the earth pressure exhibits only minor variations due to prior stress release.

When the bedrock dislocation was 2 cm (initial stage), the soil rupture conditions in Test 1 and Test 2 were generally similar, with minor localized cracking occurring at the base of the soil layer without propagating to the surface. Although only minor deformation appeared on the surface, earth pressure cells detected significant changes in the central part of the overburden (where the fault traverses). This indicates that the initial energy from bedrock dislocation was primarily consumed by generating micro-fractures within the soil mass. The geogrids effectively confined the soil, restricting upward rupture propagation, resulting in minimal earth pressure change in the upper layer.

As the bedrock dislocation increased to 5 cm (minor basal dislocation stage), the internal micro-fractures connected to form a macroscopic rupture trace that propagated through to the surface. It is noteworthy that the magnitude of earth pressure change decreased during this stage. This is because the major stress had already been released through soil yielding in the initial stage, and the current displacement was primarily converted into visible rupture and surface deformation. Comparing Test 1 and Test 2, the rupture zone in Test 2 was narrower and located farther from the structure, directly attributable to the more uniform and stronger lateral confinement provided by the additional geogrid layers.

When the bedrock dislocation reached 7 cm (final rupture stage), the aforementioned trends were further reinforced. The more pronounced confinement from the geogrids in Test 2 resulted in less developed rupture zones, and the residual earth pressure change was slightly greater than in Test 1, suggesting that the internal soil maintained higher density and stress state, indicating better structural integrity.

Comprehensive analysis indicates that earth pressure serves as an indicator of fault activity, and its evolution process, combined with the number of geogrid layers, collectively determines the final rupture pattern.

#### 3.1.2. Comparative Analysis of Surface Displacement

**Figure 9:**
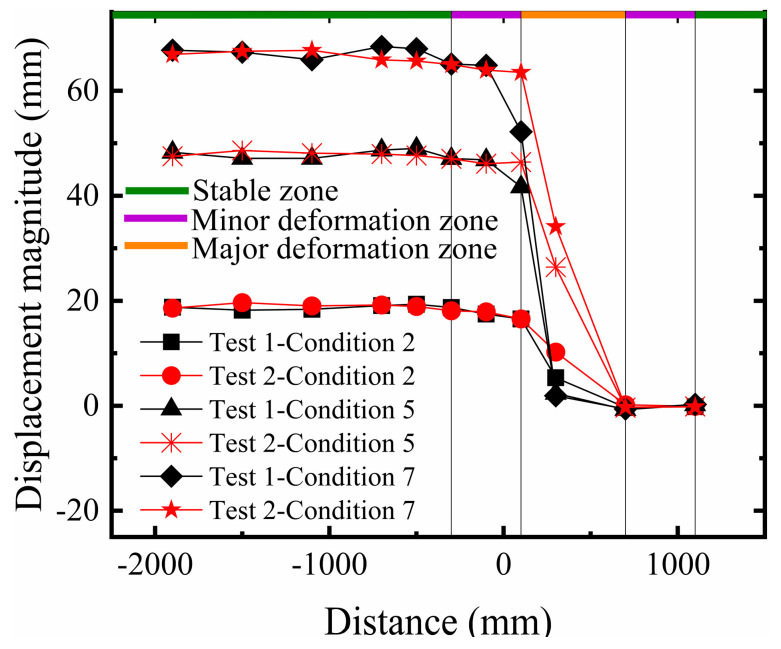
Comparison of surface displacement in sandy soil overburden.

Based on the aforementioned description of macroscopic failure phenomena, and similarly analyzing the surface deformation of the site across three failure stages, the final surface deformation characteristics can be divided into the stable zone, the minor deformation zone, and the major deformation zone. During the initial stage of fault development, the scarp was relatively gentle, and basal deformation was not significant. As the scarp gradually developed towards the final rupture stage, the extent of the steep slope expanded, and the soil near the fault in the hanging wall exhibited differential uplift. In the stable zone farther from the fault, the soil essentially underwent rigid body movement. The minor deformation zone serves as a transition area between the stable zone and the major deformation zone. With increasing dislocation, the extent of the minor deformation zone in Test 1 expanded, and the displacement amplitude increased significantly. In contrast, although Test 2 also showed an increase, the magnitude of growth was smaller, demonstrating a superior strain accommodation capacity (As shown in Figure 9). In the final rupture stage, the minor deformation zone in Test 1 expanded further with intensified displacement response, whereas in Test 2, the extent of its minor deformation zone did not increase significantly. This indicates that the multi-layer geogrid system effectively altered the transmission path of the fault displacement and the form of the surface scarp.

Overall, compared to Test 1, although the final deformation difference between the hanging wall and footwall in Test 2 was not substantial, the five-layer geogrid composite foundation further enhanced the foundation stiffness relative to the three-layer configuration, thereby improving the fracture resistance. Analysis indicates that using five geogrid layers as horizontal reinforcement can more effectively stabilize the soil and enhance structural compactness. The differential surface settlement shifted rightward overall, the surface rupture location shifted rightward overall, and the resulting step-fault scarp was located farther from the structure, reducing the impact of differential settlement on the building.

#### 3.1.3. Comparative Analysis of Microseismic Acceleration Changes in the Overburden and Structure

**Figure 10:**
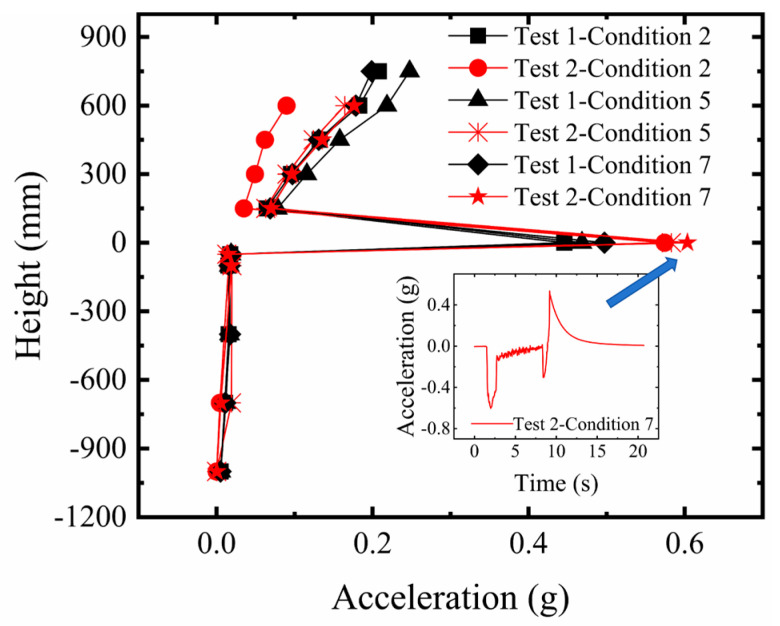
Comparison of microseismic acceleration response in sandy soil overburden and structure.

Figure 10 displays the microseismic acceleration response of the sandy soil overburden and the structure under fault pulse action. Analysis indicates that the microseismic acceleration values within the overburden remained consistently close to zero throughout the process from the initial stage to the final rupture in both Test 1 and Test 2. This suggests that the number of geogrid layers had minimal impact on the dynamic response of the overburden in this region, with their constraining effect primarily manifested in the deformation concentration zone near the fault. The microseismic acceleration showed a slight increase with decreasing thickness, but overall remained near zero. At the overburden surface, the microseismic acceleration increased with greater bedrock dislocation and intensified soil rupture, resulting in a peak pulse energy release exceeding 0.4 g. For the structure, the microseismic acceleration in the upper stories increased with building height. Under the same rupture mode, the overall acceleration response of the structure in Test 2 was smaller than in Test 1. This is attributed to the more significant soil improvement from the five-layer geogrids, which resulted in the surface rupture point being farther away, thus the structure experienced a smaller pulse response.

#### 3.1.4. Comparative Analysis of Strain in Building Edge Columns

**Figure 11:**
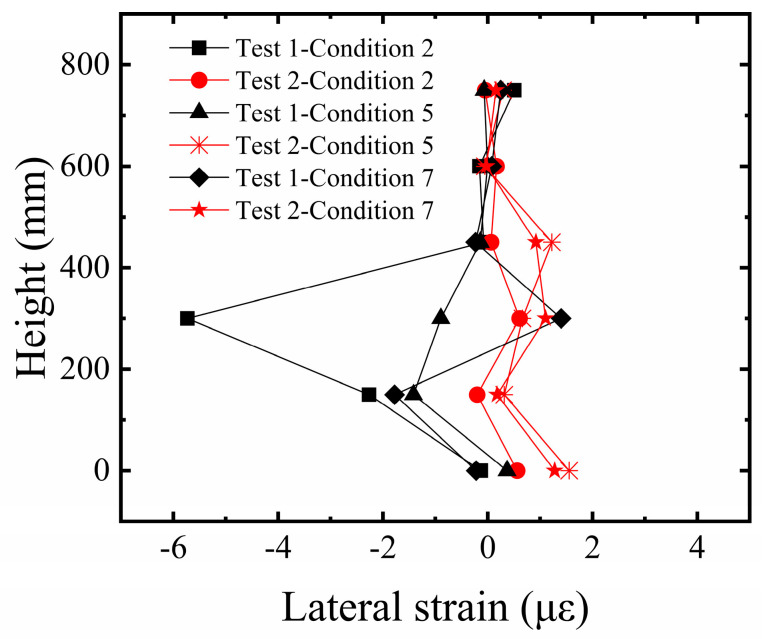
Comparison of lateral strain in edge columns of the building on sandy soil overburden.

In this study, our primary method for characterizing structural fracture resistance is by evaluating the foundation system’s ability to maintain overall stability (tilt) and control structural deformation (edge column strain) when subjected to significant surface fault rupture. The strain response of the building’s edge columns is a key indicator for revealing the structural stress state under fault displacement. As shown in Figure 11, the analysis of vertical strain in the edge columns of the building model indicates a significant regularity in the strain distribution along the height of the structure. The edge columns at the ground story exhibit the maximum strain value due to direct foundation restraint and the load transferred from the superstructure. The strain decreases progressively with increasing height, approaching zero at the top story. Furthermore, a comparison between Test 1 and Test 2 shows that the distribution trend of lateral strain along the height is generally consistent in both tests, with larger strain amplitudes in the middle and lower regions. This reflects a mechanically consistent behavior in the edge columns under different reinforcement conditions. However, the strain response in Test 1 was significantly higher than in Test 2, clearly indicating that the significant differential surface deformation, due to insufficient foundation restraint, induced severe bending deformation in the edge columns. Although the strain response of the edge columns in Test 2 increased synchronously with the bedrock dislocation, the strain amplitude was effectively controlled. This confirms that the five-layer geogrid system successfully improved the strain response of the structure under large deformation conditions.

#### 3.1.5. Strain Comparative Analysis of Three-Layer Versus Five-Layer Horizontal Reinforcement in Sand

**Figure 12:**
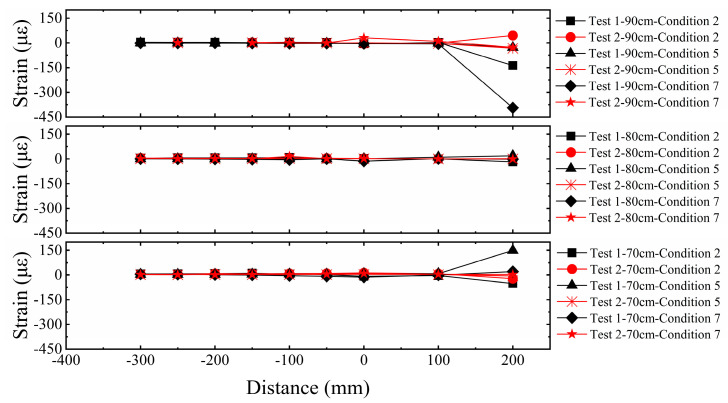
Comparison of geogrid strain in sandy soil overburden.

As shown in Figure 12, the geogrids exhibited significant strain responses within the range of the fault rupture trace, and the strain amplitude in Test 1 was markedly higher than in Test 2. This indicates that the number of geogrid layers plays a crucial role in the fracture resistance of the reinforced foundation, with fewer layers making the system more prone to larger deformations. Further comparative analysis reveals that, influenced by fault displacement, both the top and bottom layers of the geogrids showed significant strain responses, while the strain in the middle layer was relatively minor. This reflects the non-uniform stress characteristics of the reinforcement under fault action. Furthermore, the rupture deformation was primarily concentrated at the ground surface and the bedrock rupture point, while the middle soil layer tended to become denser due to the influence of the horizontal reinforcement. As the number of geogrid layers increased, the overall stiffness and bearing capacity of the composite foundation were significantly enhanced, thereby effectively suppressing the deformation of the geogrids themselves in Test 2. This mechanism strengthened the composite foundation’s resistance to surface rupture, enabling it to provide a superior surface scarp pattern and enhanced stability for the superstructure in fault outcrop areas.

### 3.2. Comparative Analysis of Sand Site Versus Clay Site with Three-Layer Horizontal Reinforcement

#### 3.2.1. Comparative Analysis of Fault Rupture

**Figure 13:**
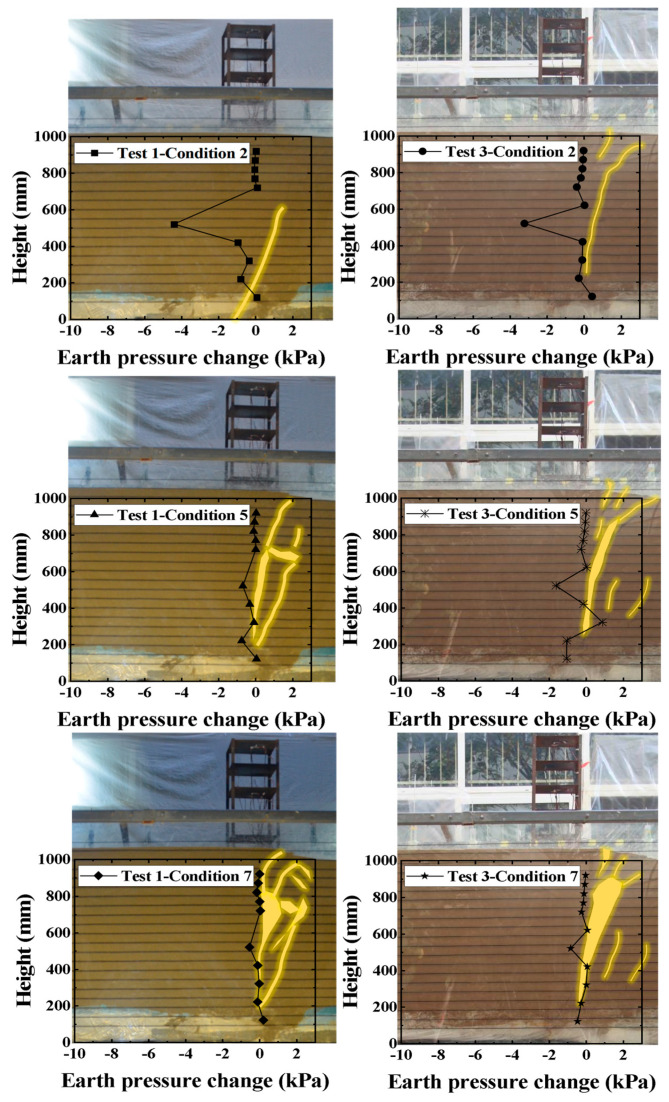
Diagram of earth pressure variation within the overburden and the development pattern of the rupture trace (Comparison of Test 1 and Test 3).

Figure 13 presents the macroscopic rupture phenomena and the corresponding internal earth pressure changes for Test 1 and Test 3 at bedrock dislocation amounts of 2 cm, 5 cm, and 7 cm. The test results indicate that the degree of differential surface deformation and the development of the internal soil fault progressively intensified with increasing bedrock dislocation.

During the initial stage of bedrock dislocation, no significant fault rupture signs were observed in Test 1 and Test 3, and no noticeable tilting occurred in the building. The magnitude of earth pressure change within the overburden and its variation trends were generally consistent between Test 1 and Test 3, and highly consistent with the fault rupture path. Test 3 exhibited a smaller earth pressure response during the initial stage, indicating that the composite foundation formed by the horizontal geogrid reinforcement and the soil in the clay site possesses superior fracture resistance, maintaining higher structural stability when subjected to fault displacement.

When the bedrock dislocation increased to 5 cm, significant differential deformation appeared on the surface. The soil in Test 1 was looser with a wider rupture extent compared to Test 3, while the clay exhibited a wider main rupture zone with more pronounced rupture phenomena. The rupture path in the clay site deviated significantly from that in the sand site, causing the fault trace to move away from the structure and form a step-fault scarp. It is noteworthy that during this stage, the magnitude of earth pressure change in Test 3 was greater than in Test 1, indicating that the clay site maintained a stronger capacity to resist fault displacement even after undergoing prior dislocation.

When the bedrock dislocation reached the final rupture stage, the clay site demonstrated a more pronounced soil improvement effect due to the stronger interaction between the foundation and the horizontal reinforcement. This is specifically manifested by the fact that the magnitude of earth pressure change in Test 3 remained higher than in Test 1 during this stage. Macroscopically, the step-fault scarp was located farther from the building foundation, and the structure maintained relative stability without significant tilting. This indicates that the clay site still retained a certain potential for fracture resistance.

#### 3.2.2. Comparative Analysis of Surface Displacement

**Figure 14:**
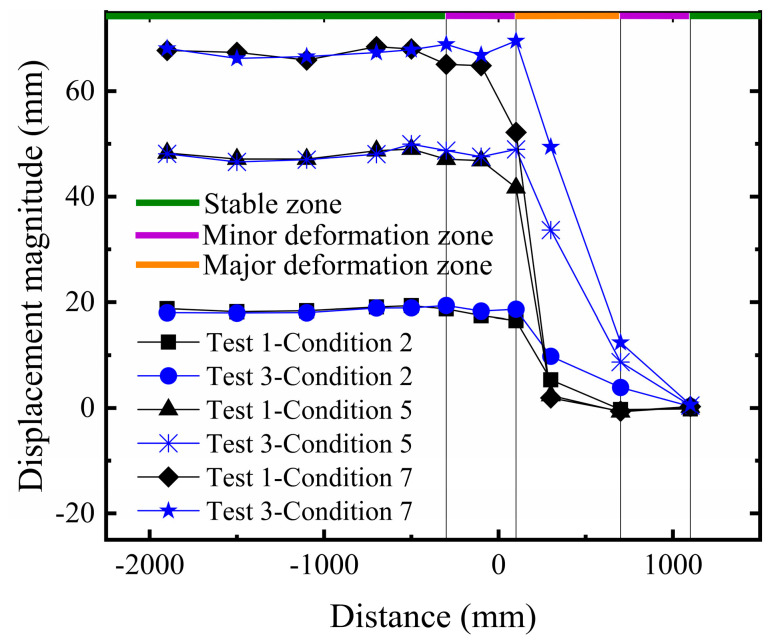
Comparison of surface displacement in clay overburden.

As shown in Figure 14, comparing the surface deformation characteristics of Test 1 and Test 3, the soil in the stable zone still maintained rigid body movement. With increasing bedrock dislocation, both the minor and major deformation zones in Test 3 shifted rightward overall, and the step-fault scarp developed more distinctly. This step-fault scarp is more conducive to ensuring the safety of the structure in the clay site, indicating that with the same number of geogrid layers, superior fracture resistance performance can be achieved in clay, providing better control over the significant surface deformation induced by the fault.

#### 3.2.3. Comparative Analysis of Microseismic Acceleration Changes in the Overburden and Structure

**Figure 15:**
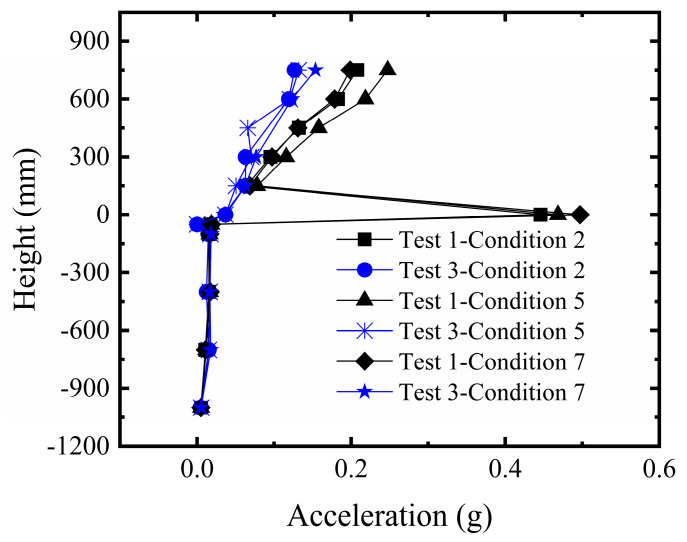
Comparison of microseismic acceleration response in clay overburden and structure.

As shown in Figure 15, significant differences in the microseismic response at the overburden surface and the building’s ground story were observed between Test 1 and Test 3. In the sand site, the pulse wave effect generated by the energy release during fault rupture induced larger microseismic accelerations. In contrast, the clay site, leveraging the cohesive strength of the soil and the synergistic interaction with the geogrids, enhanced the integrity of the composite foundation. This resulted in reduced energy release during soil rupture, leading to a weaker monitored microseismic acceleration response. The microseismic accelerations on all stories of the building in Test 1 were greater than those in Test 3, further confirming that the dynamic impact of fault rupture is more pronounced in the sand site.

#### 3.2.4. Comparative Analysis of Strain in Building Edge Columns

**Figure 16:**
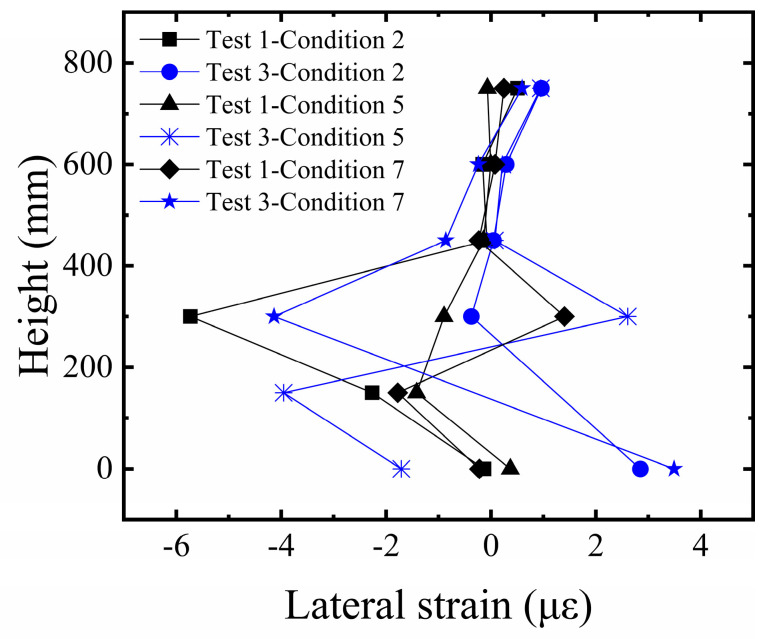
Comparison of lateral strain in edge columns of the building on clay overburden.

By comparing the lateral strain in the building’s edge columns (Figure 16), the lateral strain is the smallest in the upper edge columns, shows minor fluctuations in the lower part, and is most significant in the middle part. The lateral strain values in the sand site are generally greater than those in the clay site, indicating that the geogrid reinforcement provides superior improvement in the clay site, effectively reducing the impact of fault activity on the superstructure response.

#### 3.2.5. Strain Comparative Analysis of Three-Layer Horizontal Reinforcement in Sand Versus Clay

**Figure 17:**
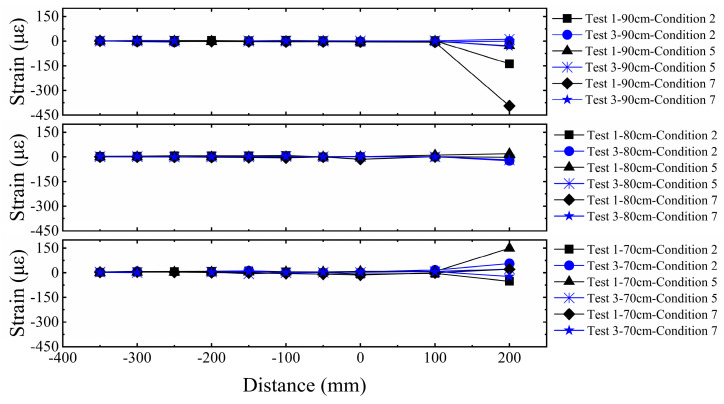
Comparison of geogrid strain in clay overburden.

Figure 17 presents the strain distribution of the geogrids, showing significant strain variation at the fault crossing location. The strain amplitude in the geogrids within the sand site is markedly greater than in the clay site, indicating stronger soil-reinforcement interfacial interaction in the clay and superior fracture resistance of the composite foundation. Influenced by the fault displacement, both the top and bottom layers of the geogrids exhibited larger strains in both sites, whereas the strains in the middle layer were relatively minor. This recurring phenomenon reveals that the top and bottom geogrid layers bear the primary deformation induced by the fault, while the middle layer primarily functions to coordinate the deformation. Therefore, in practical anti-fracture engineering, enhancing the structural measures for the top and bottom geogrid layers could be considered to improve the overall system’s disaster resistance and mitigate the hazard of fault displacement to the superstructure. Based on the experimental results, we conducted a comparative analysis of the seismic damage severity across the three sets of tests, and the findings are summarized in Table 5.

## 4. Failure Mechanism

To further reveal the working mechanism of geogrid-reinforced composite foundations under normal fault displacement, this section systematically elucidates, based on experimental observations and data analysis, how different soil properties influence the synergistic performance of the “soil-reinforcement” system and the resulting macroscopic failure mode. The mechanistic analysis in this section will provide a solid theoretical basis for the fracture-resistant design of horizontal reinforcements in engineering structures crossing normal faults.

During the initial stage of bedrock dislocation, although no noticeable cracks appeared on the surface, deep earth pressure cells had already detected significant stress changes, and the development of rupture was clearly observed as the bedrock dislocation gradually increased. This specific experimental phenomenon confirms that deep stress changes possess a distinct precursor characteristic relative to fault development. Conversely, the surface microseismic acceleration remained largely unchanged during the initial stage but exhibited a pulse peak exceeding 0.4 g when surface rupture occurred, indicating it can serve as an indicator of disaster onset. This analysis, based on the differences across specific experimental phases, provides a solid physical foundation for building a real-time monitoring system with sensors deployed deep within the overburden.

Under normal fault displacement, the geogrids installed in the sandy soil overburden altered the rupture mode and deformation characteristics of the soil mass. Within the fault crossing zone, the geogrids effectively dispersed the primary fault rupture over a wider soil area, thereby suppressing the severe development of surface rupture. Specifically, the middle and lower geogrid layers participated in deformation coordination, enhancing the integrity and shear resistance of the soil mass, and guiding the rupture plane to propagate away from the structure. The upper geogrid layers primarily sustained tensile stress, delaying the connection of surface cracks and effectively altering the location of the fault outcrop. Ultimately, the soil-reinforcement interaction transformed the potentially formed plastic shear deformation zone into a concentrated, structure-distant “V”-shaped rupture zone and a step-fault scarp at the surface, significantly improving the foundation’s fracture resistance and deformation compatibility under fault displacement.

In the clay overburden, the presence of cohesion between the geogrids and the soil results in the formation of a stiffer fracture-resistant system, strengthening the synergistic working mechanism of the “overburden soil-horizontal reinforcement”. Within this system, the geogrids effectively suppress the propagation of near-surface cracks by resisting tensile stresses, while the lower geogrid layers, together with the surrounding clay, form an integrated shear-resistant zone, forcing the fault rupture path to deviate and altering the fault outcrop location and surface deformation pattern. Unlike in the sand site, the rupture zone in the clay exhibits a banded distribution, and the slope of the differential surface deformation zone is gentler. This reflects the superior ability of clay to absorb and dissipate the energy from fault displacement, significantly enhancing the cracking resistance of the composite foundation under fault movement.

**Figure 18:**
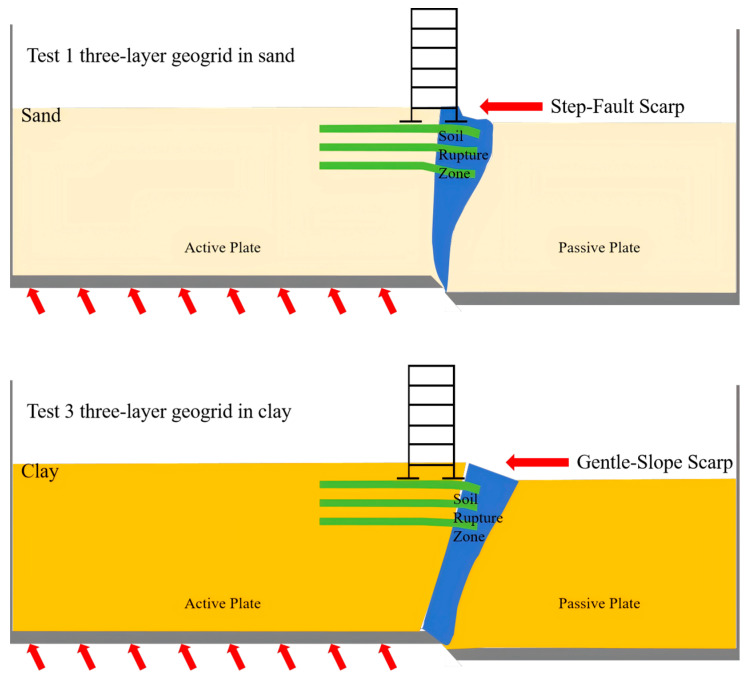
Schematic diagram of the rupture mechanism in geogrid-reinforced site.

As shown in Figure 18, the core innovation of this horizontally reinforced composite foundation lies in its mechanism that actively transfers the energy induced by the fault. Unlike traditional rigid foundations, the multi-layer geogrid system utilizes the soft tensile resistance of the grids themselves to counteract the shear failure caused by the fault, thereby enhancing the shear strength of the foundation soil. This confinement causes the fault rupture to deviate towards weaker zones of the foundation soil and successfully alters the location and morphology of the surface rupture. Consequently, it effectively transforms the surface deformation pattern from a destructive gentle slope into a safer step-fault scarp located farther from the structure.

## 5. Conclusions

This study established large-scale, complex geomechanical models for both sand and clay sites, integrating displacement meters, earth pressure cells, and accelerometers. It developed a comprehensive “fault rupture–foundation response–structural performance” analytical framework, deepened the understanding of soil-structure interaction mechanisms, and revealed the evolution patterns of internal dynamic responses within the foundation during fault displacement, providing novel data for mechanistic understanding. The specific conclusions are as follows:

1. The differential surface deformation is significantly influenced by the foundation stiffness. The five-layer geogrid configuration notably enhances the lateral stiffness of the foundation, successfully transforming the broad “V”-shaped graben within the major deformation zone into a safer step-fault scarp. This mechanism effectively restricts the width of the rupture zone and forces the rupture path to deviate significantly to the right, directing it away from the building foundation area, thereby mitigating the impact on structural safety.

2. Earth pressure serves as a precursor key indicator for revealing the fault rupture process, with its significant changes consistently preceding the development of visible rupture. During the initial stage of bedrock dislocation, although no obvious surface cracks appeared, deep earth pressure cells had already detected significant stress changes, confirming that deep stress variations possess a distinct precursor characteristic relative to fault development. Comparing the earth pressure changes across the three sets of tests, the cohesive soil site demonstrated the most pronounced fracture resistance, whereas the sand site with three-layer geogrids showed deficiencies in its anti-fracture performance.

3. Under identical geogrid configurations, the cohesion of clay significantly enhances the interfacial interaction with the geogrids, resulting in greater overall integrity. Consequently, the fracture resistance performance of the clay site is comprehensively superior to that of the sand site. This indicates that increasing the number of geogrid layers provides stronger and more uniform horizontal confinement, thereby enhancing the overall stiffness of the composite foundation. These findings provide an important basis for optimizing the fracture-resistant design of horizontal reinforcement systems.

4. Future work should consider applying bedrock dislocation while extending the investigation to multiple dislocation modes such as reverse faults and strike-slip faults, to further explore fracture-resistant design theories applicable to different fault types and soil conditions.

In summary, this study deepens the understanding of the mechanisms by which horizontally reinforced composite foundations mitigate fault displacement and large surface deformation under normal faulting and provides experimental evidence for the seismic design and disaster mitigation of engineering structures in near-fault regions. Future research will build upon addressing these limitations and expand towards more realistic and complex working conditions.

## Figures and Tables

**Table 1:** Soil parameters and their similitude constants in the model.

Physical Quantity	Similitude Constant
*l*: Length	*C_l_* = 20
*ρ*: Density	*C_ρ_* = 1
*g*: Gravity	*C_g_* = 1
*τ*: Earth Pressure	*C_τ_ = C_l_·C_ρ_·C_g_* = 20
*u*: Displacement	*C_u_* = 20

**Table 2:** Building model parameters and their similitude constants.

Physical Quantity	Similitude Constant
*l*: Length	*C_l_* = 20
*ρ*: Density	*C_ρ_* = 1
*g*: Gravity	*C_g_* = 1
*σ*: Stress	*C_τ_ = C_l_·C_ρ_·C_g_* = 20
*ε*: Stress	*C_ε_* = 1
*a*: Building Acceleration	*C_a_* = 1
*t*: Acceleration Duration	*C_t_* = $\sqrt{C_{l}/C_{g}}$ = 4.47

**Table 3:** Different test schemes.

Test Number	Number of Reinforcement Layers	Reinforcement Spacing (mm)	Soil Type	Fault Dip Angle	Fault Type	Bedrock Dislocation
1	3	100	Sand	70°	Normal Fault	1000 mm
2	5	50	Sand	70°	Normal Fault	1000 mm
3	3	100	Clay	70°	Normal Fault	1000 mm

**Table 4:** Sensor parameters table.

No.	Category	Model	Parameters
1	Strain sensor	BX120-5AA (Quzhou City Kecheng Chenxing Electronic Business Service Department, Quzhou, China)	1. Size: 5 mm × 3 mm; 2. Sensitivity: 2.08 ± 1%.
2	Earth Pressure Sensor	ESP-II (Qizhou City Kechen District Chenfeng Electronic Products Trading Department, Qizhou, China)	Range: 50 kPa; Accuracy: 0.5% FS; Measurement Type: Voltage Measurement; Sampling Frequency: 0–100 Hz
3	Jacking Displacement Sensor	YHD-200 (Liyang City Instrument Factory, Liyang, China)	Range: 0–200 mm; Bridge Configuration: Full-bridge or Half-bridge; Accuracy: 0.05 mm; Sampling Frequency: 0–50 Hz
4	Microseismic Sensor	DCIEM-M (Beidaihe District Yijia Sensor Factory, Beidaihe, China)	Range: ±1 g; Accuracy: 0.1%; Measurement Type: Voltage Measurement; Sampling Frequency: 0–100 Hz

**Table 5:** Building model parameters and their similitude constants.

Key Indicator	Test 1	Test 2	Test 3
Surface Rupture Pattern	Wide zone; large deformation.	Narrow zone; Step-Fault Scarp.	Banded; Gentle-Slope.
Rupture Path Deviation	Close to structure	Deviated farther	Deviated significantly
Structural Acceleration	Highest	Moderate	Lowest
Lateral Strain in Columns	Highest	Moderate	Lowest
Geogrid Strain	Highest	Lowest	Moderate

## Data Availability

The data that support the findings of this study are available from the first author upon reasonable request.
